# 

**Published:** 1890-11

**Authors:** 


### CONTENTS FOR NOVEMBER, 1890.

ORIGINAL COMMUNICATIONS.

Certain Causes of Major Pelvic Troubles Traceable to Minor Gynecology. By JOSEPH PRICE, M. D .......... 193
Hematoma Auris. By B. H. GROVE, M. D. .......... 205
The Muscles of the Fore-arm. By A. L. BENEDICT, M. D .......... 211

SOCIETY PROCEEDINGS.

Philadelphia County Medical Society .......... 214
American Association of Obstetricians and Gynecologists .......... 220
Virginia Medical Society .......... 224

PROGRESS IN MEDICAL SCIENCE.

Physiological Chemistry. By JOHN A. MILLER, A. M., Ph. D .......... 225
Nervous Diseases, Insanity and Bacteriology. By WILLIAM C. KRAUSS, M. D .......... 230
The Influence of Electricity on Protoplasm .......... 232
Ophthalmoscopic Examination of the Eyes of the Hypnotized .......... 233
New Methods of Treatment in Erysipelas .......... 234

CORRESPONDENCE.

Dr. Hayd Replies to J. P. and J. H .......... 236
The Hospital Graduate Degree .......... 240

LITERARY NOTES.

Seventh Edition "Da Costa" .......... 242

EDITORIAL.

The New Jersey State Examining Board .......... 243
Another New Medical Law .......... 244
Meeting of the Mississippi Valley Medical Association .......... 245
Society Meetings .......... 246
Personal .......... 247
Obituary .......... 248
Journalistic Notes .......... 249
Reviews .......... 250

